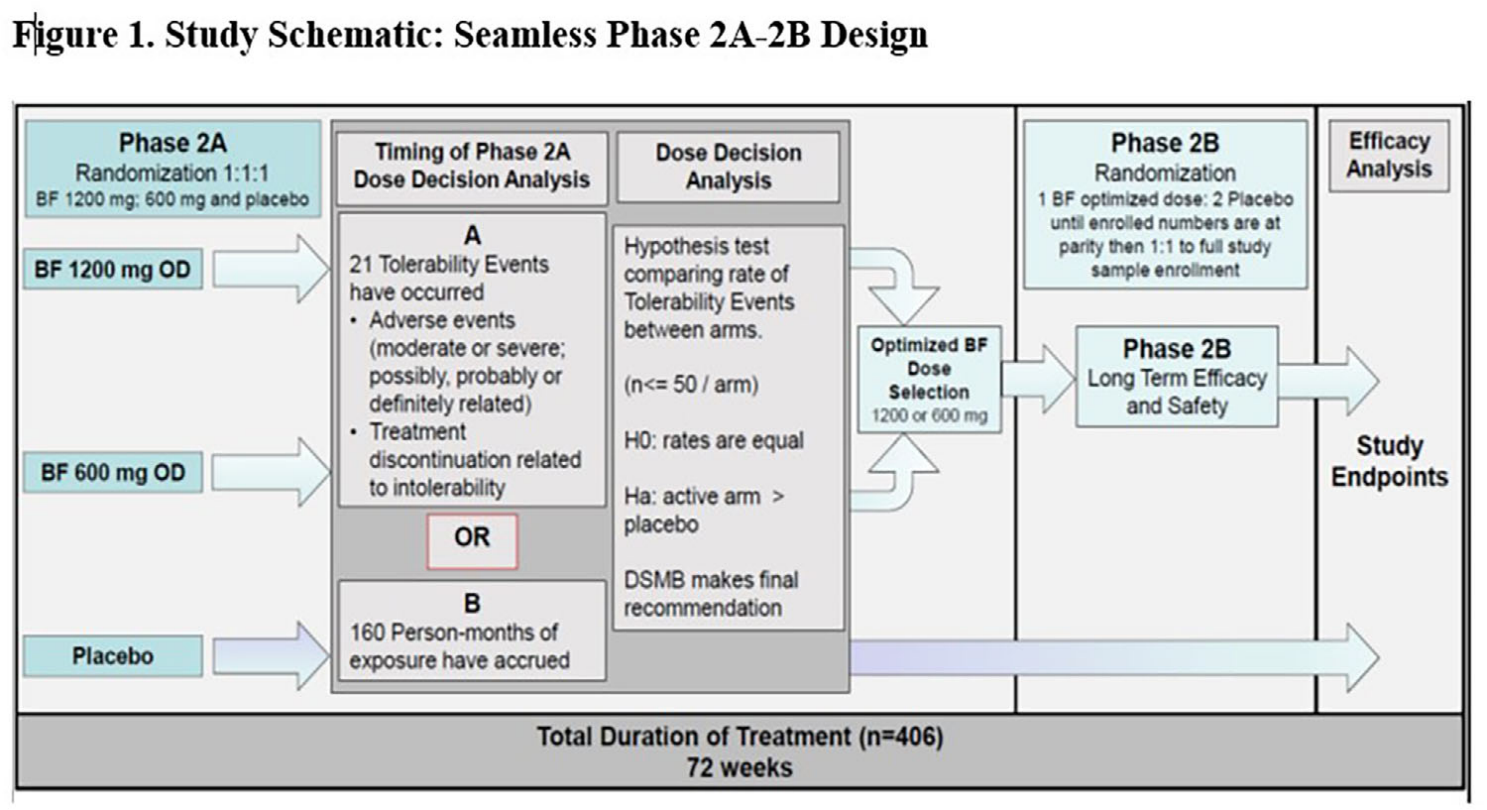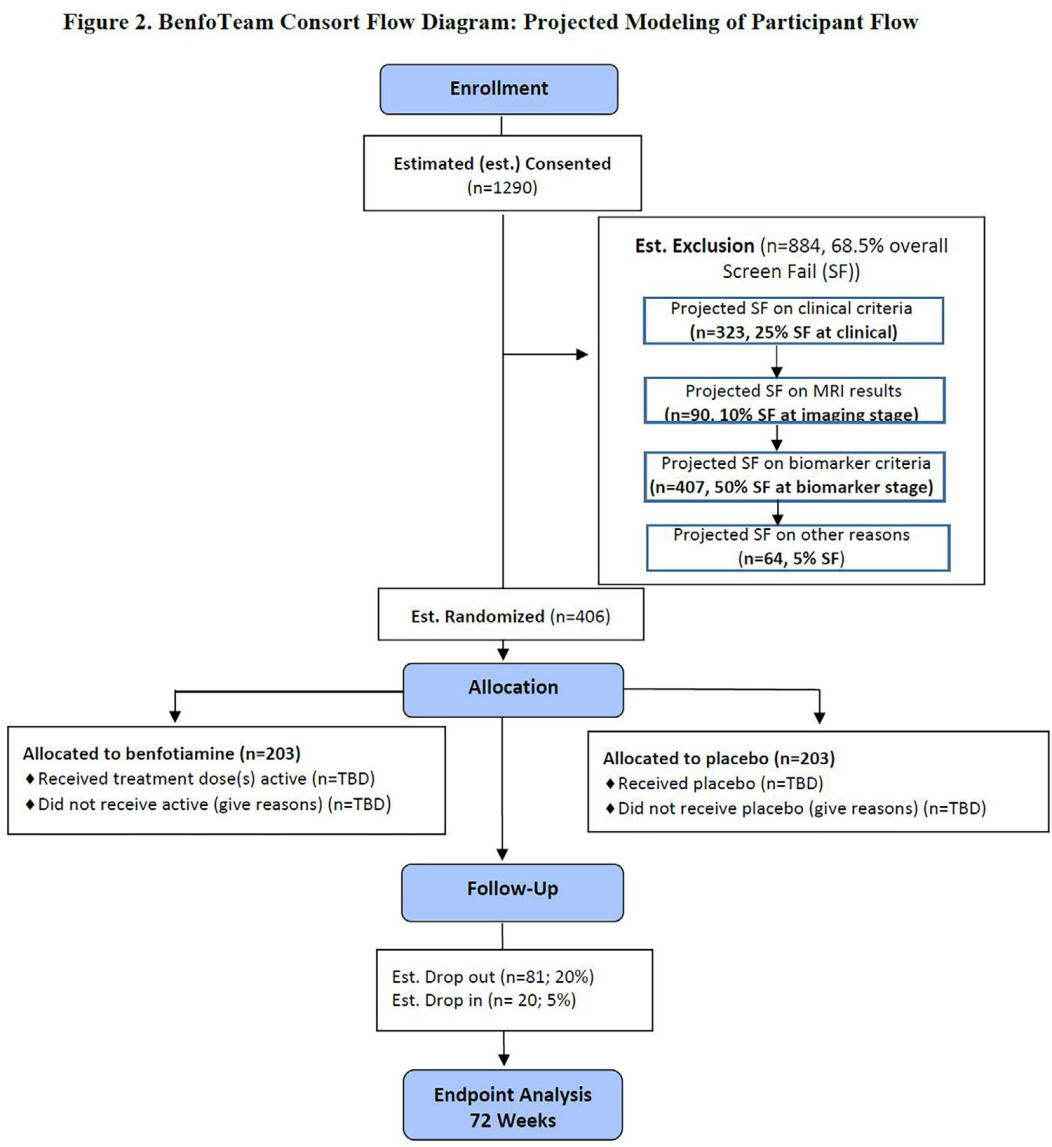# A Seamless Phase 2A‐Phase 2B Multi‐Center Trial to Test the Benefits of Benfotiamine on the Progression of Alzheimer’s Disease‐Benfoteam: Design and Methods

**DOI:** 10.1002/alz.091963

**Published:** 2025-01-09

**Authors:** Jose A. Luchsinger, Howard H. Feldman, Karen Messer, Steven D. Edland, Gabriel C Leger, Diane M. Jacobs, David P. Salmon, Carolyn Revta, Jody‐Lynn Lupo, January Durant, Gary E. Gibson

**Affiliations:** ^1^ Columbia University Irving Medical Center, New York, NY USA; ^2^ University of California San Diego, La Jolla, CA USA; ^3^ Burke Neurological Institute, Weill Cornell Medicine, White Plains, NY USA

## Abstract

**Background:**

Benfotiamine, a prodrug of thiamine, raises blood levels by 50‐100 times to achieve pharmacologic effects. It provides a novel therapeutic direction addressing a well‐characterized brain tissue thiamine deficiency and related changes in glucose metabolism in AD. BenfoTeam is a seamless phase 2A‐2B “proof of concept” (POC), double‐blind, placebo‐controlled RCT investigating tolerability, safety, and efficacy of benfotiamine, as a first‐in‐class small molecule treatment for early AD.

**Method:**

Participants (n = 406) with MCI or mild dementia due to AD with a positive plasma biomarker test result (C2N PrecivityAD2) will be enrolled at ∼50 sites in the U.S. Phase 2A will evaluate safety and tolerability to determine which dose (600 mg or 1200 mg) to carry forward into phase 2B. This dose decision will be determined at the first of either 21 total adverse tolerability events (TEs) or alternatively when 160 person‐months of exposure have accumulated (see Figure 1). If 1200 mg/day does not have a significantly higher TE rate than placebo, then this dose will be taken forward to phase 2B and tested against placebo without stopping the trial, otherwise 600 mg/day will be carried forward. Phase 2B will assess efficacy and longer‐term safety of benfotiamine in the full study sample through 72 weeks of treatment, at the selected dose.

**Result:**

Our trial modeling is presented in Figure 2. We project that to achieve our total sample size of 406 (203/arm), ∼ 1290 participants will need to be screened. With a 20% dropout rate, and a 5% drop‐in rate from the placebo arm to active treatment, the trial will be powered at 80% overall (90% on each endpoint separately) to detect a difference between arms on co‐primary endpoints (CDR‐SB and ADAS‐Cog 13) at 72 weeks, corresponding to a Cohen’s D effect size of 0.38 (small to moderate effect size).

**Conclusion:**

BenfoTeam deploys an innovative seamless phase 2A‐2B design to achieve its overall aim of clinical POC. With its early adaptive dose decision, exposure will be optimized to the highest tolerated dose. A positive phase 2 POC trial that is powered to achieve a clinically significant benefit will further validate our approach.